# ­­­­­Comparison of T1-Post and FLAIR-Post MRI for identification of traumatic meningeal enhancement in traumatic brain injury patients

**DOI:** 10.1371/journal.pone.0234881

**Published:** 2020-07-02

**Authors:** Tara S. Davis, Jennifer E. Nathan, Ana S. Tinoco Martinez, Jill B. De Vis, L. Christine Turtzo, Lawrence L. Latour

**Affiliations:** 1 Center for Neuroscience and Regenerative Medicine, Bethesda, Maryland, United States of America; 2 Johns Hopkins Suburban Hospital, Bethesda, Maryland, United States of America; 3 National Institute of Neurological Disorders and Stroke, National Institutes of Health, Bethesda, Maryland, United States of America; Medical University of Vienna, AUSTRIA

## Abstract

Traumatic meningeal enhancement (TME) is a novel biomarker observed on post-contrast fluid-attenuated inversion recovery (FLAIR) in patients who undergo contrast-enhanced magnetic resonance imaging (MRI) after suspected traumatic brain injury (TBI). TME may be seen on acute MRI despite the absence of other trauma-related intracranial findings. In this study we compare conspicuity of TME on FLAIR post-contrast and T1 weighted imaging (T1WI) post-contrast, and investigate if TME is best detected by FLAIR post-contrast or T1WI post-contrast sequences. Subjects selected for analysis enrolled in the parent study (NCT01132937) in 2016 and underwent contrast-enhanced MRI within 48 hours of suspected TBI. Two blinded readers reviewed pairs of pre- and post-contrast T1WI and FLAIR images for presence or absence of TME. Discordant pairs between the two blinded readers were reviewed by a third reader. Cohen’s kappa coefficient was used to calculate agreement. Twenty-five subjects (15 males, 10 females; median age 48 (Q1:35-Q3:62; IQR: 27)) were included. The blinded readers had high agreement for presence of TME on FLAIR (Kappa of 0.90), but had no agreement for presence of TME on T1WI (Kappa of -0.24). The FLAIR and T1WI scans were compared among all three readers and 62% of the cases positive on FLAIR could be seen on T1WI. However, 38% of the cases who were read positive on FLAIR for TME were read negative for TME on T1WI. Conspicuity of TME is higher on post-contrast FLAIR MRI than on post-contrast T1WI. TME as seen on post-contrast FLAIR MRI can aid in the identification of patients with TBI.

## Introduction

In 2013 approximately 2.8 million emergency department visits, hospitalizations, and deaths in the United States resulted from traumatic brain injury (TBI) [1]. Annually, approximately 75% of TBIs presenting at the hospital are mild in severity, with post-traumatic loss of consciousness, amnesia, or confusion lasting less than 1 hour in duration [2]. Despite its terminology, mild TBI (mTBI) poses a major health concern as over 30% of patients still experience functional impairment at 3 months follow-up [3]. Unfortunately, mTBI patients may not be adequately diagnosed at initial presentation [4] regardless of meeting criteria for mTBI based on the American Congress of Rehabilitation Medicine [5].

**Fig 1:**
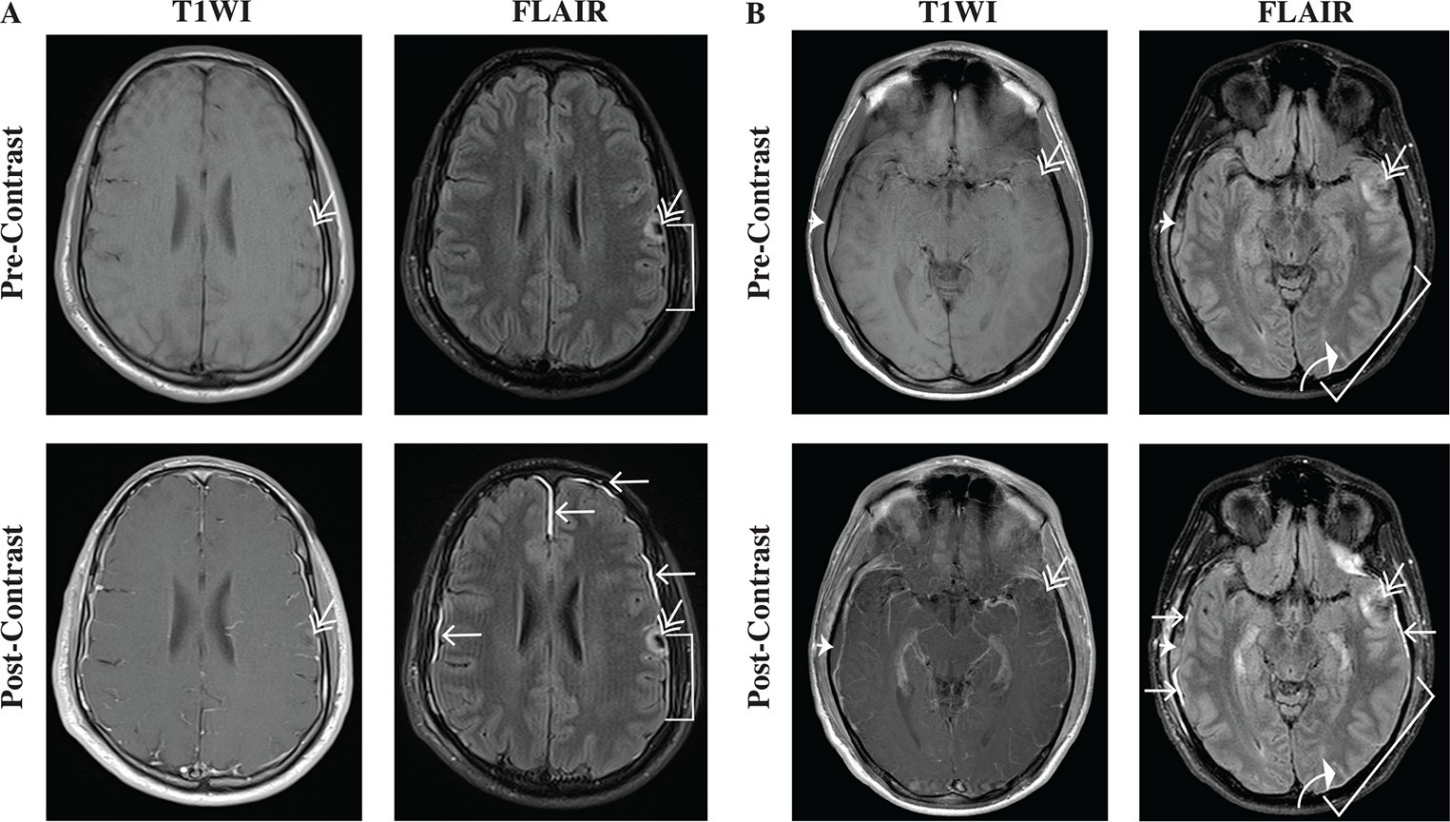
T1WI and FLAIR pre- and post-contrast MRI can reveal different types of TBI-related pathology in patients presenting acutely (< 48 hours) after injury. Radiological conventions are used here, with the patient’s left side on the right-hand side of the image. (A) The first series of images shows a representative example of a TBI patient with a contusion (double arrow), a subdural hematoma (bracket), and traumatic meningeal enhancement (TME) (single arrows). On both T1WI pre- and post-contrast images, the small contusion on the patient’s left side is more noticeable on FLAIR images secondary to associated edema. The pre-contrast and post-contrast FLAIR demonstrate a thin subdural hematoma adjacent to the contusion, which is not readily apparent on the T1WI images. On post-contrast FLAIR, areas of TME are also evident. In comparison, the T1WI post-contrast imaging reveals mostly vascular patterns in the parenchyma and along the meninges. (B) The second series of images shows images from another representative TBI patient with a small contusion (double arrow), a small epidural hematoma (thick arrowhead), a thin subdural hematoma (bracket), and associated subarachnoid hemorrhage (curved white arrow), in addition to TME (single arrows).

Most medical centers continue to use CT imaging as a standard imaging modality when mTBI is suspected, even though it has a low sensitivity for detecting any evidence of mild injury [6, 7]. A number of comparative CT-MRI studies have shown acute findings that are not visualized on CT but are detected on MRI in mTBI [8, 9]. MRI can provide additional insights into pathology in acute TBI patients, as is seen in these examples from research patients in our large longitudinal natural history study, the Traumatic Head Injury Neuroimaging Classification (THINC) study (Fig 1).

Enhancement of the meninges is observed on FLAIR post-contrast imaging in approximately 1 out of 2 research patients with suspected TBI who are scanned within 48 hours of sustaining a head injury [8, 10]. This novel biomarker is called traumatic meningeal enhancement (TME) and is thought to result from the extravasation of contrast into the subdural space due to trauma that separates the arachnoid mater from the dura mater [10]. The pattern of findings on T1WI and FLAIR pre- and post-contrast may appear different, raising the question as to which sequence is better for detecting TME.

Although the clinical significance of TME is not yet known, it may be a useful imaging marker for the diagnosis of TBI. While the utility of FLAIR post-contrast imaging is under evaluation in TBI research studies [8, 10, 11], T1WI post-contrast images are more often obtained in clinical practice [12]. The purpose of this retrospective study is to compare the conspicuity of TME findings on FLAIR post-contrast versus T1WI post-contrast images to determine which sequence(s) are best for identification of this new imaging biomarker of TBI and to distinguish it from TBI-related pathology seen on other MRI sequences.

## Materials and methods

### Patient population

**Fig 2:**
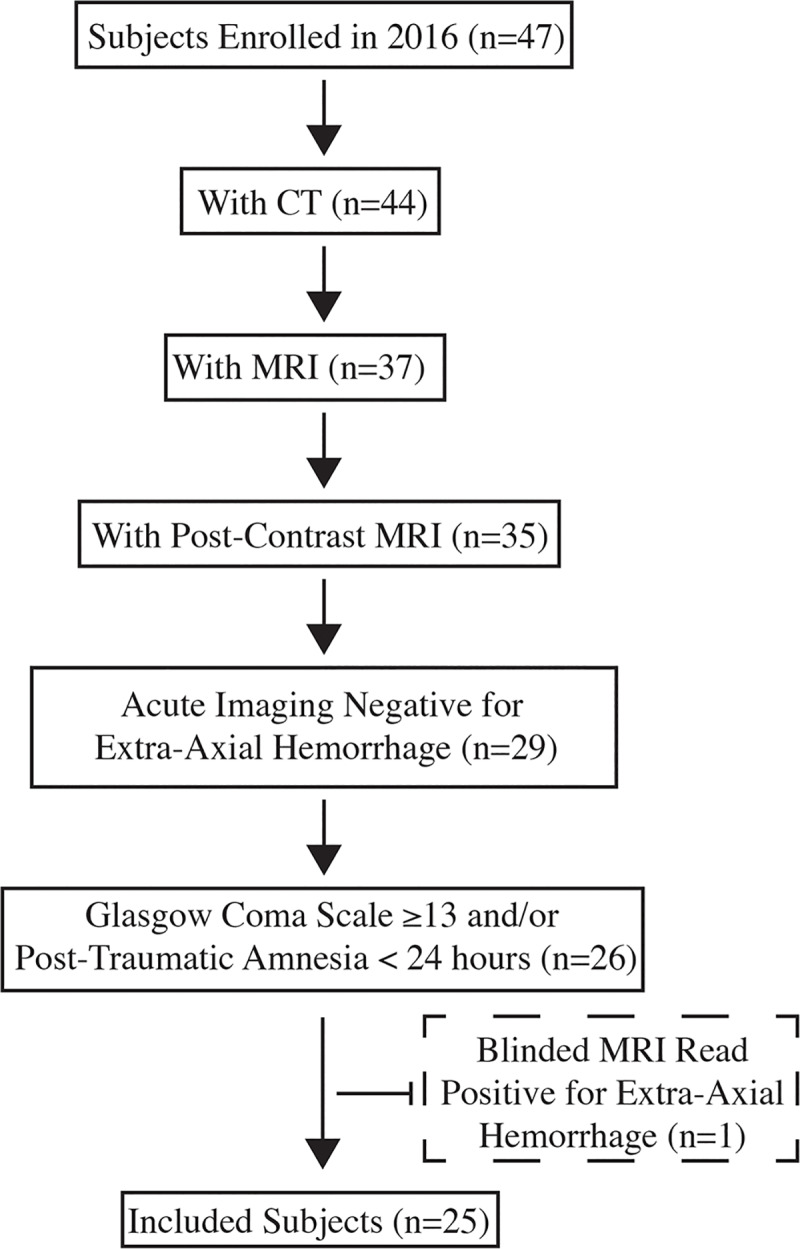
Flowchart demonstrating how the final inclusion set was obtained. Extra-axial hemorrhage was defined as subarachnoid hemorrhage, subdural hematoma, and/or epidural hematoma.

This protocol was approved by the National Institutes of Health Intramural Institutional Review Board and written informed consent was obtained prior to any study procedures. Subjects for this analysis were selected from consented patients who enrolled into the Traumatic Head Injury Neuroimaging Classification (THINC) study (NCT01132937) at a Level II trauma center in the Washington DC metropolitan area during the year 2016. The THINC study includes time points out to 1 year post-injury. Specific inclusion criteria for the analysis performed in this subanalysis of the THINC study included: 1) 18 years of age or older, 2) MRI completed within 48 hours of suspected head injury, 3) received a single dose of gadolinium-based contrast agent during MRI, 4) negative clinical radiology CT and MRI report for extra-axial hemorrhage (subarachnoid hemorrhage (SAH), subdural hematoma (SDH), or epidural hematoma (EDH), 5) research MRI image protocol included pairs of pre- and post-contrast T1WI and FLAIR sequences, 6) initial Glasgow Coma Scale (GCS) of ≥13 assessed in the emergency department, loss of consciousness length of time <30 minutes, and post-traumatic amnesia (PTA) length of time <24 hours. Reasons for subject exclusion were as follows: 1) clinical CT not completed, 2) research MRI with contrast not completed, 3) acute imaging positive for SAH, SHD, and/or EDH, 4) GCS <13 and/or PTA >24 hours, 5) SDH found on blinded read (Fig 2).

### Imaging protocol

MRI was performed for research purposes on a Siemens Magnetom Skyra clinical 3T MRI scanner (Siemens Healthcare; Malvern, PA) with an inner diameter of 70 cm, using a 20 channel head and neck coil. The total acquisition time for the MRI protocol was under thirty minutes and included the following sequences acquired in the following order: DTI, T2* gradient recalled echo, SWI, three-dimensional spoiled gradient recalled T1WI, pre-contrast T1WI, pre-contrast FLAIR, dynamic contrast-enhanced EPI FLAIR, post-contrast T1WI and post-contrast FLAIR. A single weight adjusted dose (0.1 mmol/kg) of the macrocyclic gadolinium-based contrast agent gadobenate dimeglumine (Bracco Diagnostics, Monroe Township, NJ) was administered immediately prior to the start of the dynamic contrast-enhanced FLAIR. The T1WI images were acquired with 550/9 ms (TR/TE) and 2 averages while the FLAIR were acquired with 9000/120/2500 ms (TR/TE/TI) and 1 average. Both sequences had similar resolution: 178 x 220 mm field of view, 40 contiguous 3.5 mm axial oblique slices.

### Blinded image interpretation

Blinded to the subject’s identity and clinical history, two trained radiologists (a board-certified neuroradiologist and a third-year radiology resident) reviewed pairs of T1WI pre- and post-contrast and FLAIR pre- and post-contrast images for presence or absence of TME. Readers were first given the pairs of T1WI pre- and post-contrast images to review; one to two weeks later readers reviewed the pairs of FLAIR pre- and post-contrast images. In cases of disagreement between readers on the scoring of images, tie-breaking reads were completed by a trained neurologist. To assess intra-rater reliability, at least one month after initial readings the images were re-blinded and read again by both blinded readers.

### Outcome measures

The outcome measures for this study were to evaluate and identify post-contrast findings on T1WI and FLAIR, focusing on characteristics of TME, if observed.

### Statistical analysis

Contingency tables (2x2) were constructed to analyze reading results. Cohen’s Kappa coefficient was calculated for inter-rater reliability as well as intra-rater reliability, and Kappa was interpreted using the Landis and Koch benchmark scale. Values <0 were considered to reflect poor agreement; 0 to 40, slight to fair agreement; 0.41 to 0.80, moderate to substantial agreement; and 0.81–1.00, almost perfect agreement [13].

### Subtraction imaging

To better visualize areas of enhancement, pre-contrast were subtracted from post-contrast imaging. For T1 and FLAIR sequences, pre-contrast images first underwent mid-sagittal alignment in MIPAV (Medical Image Processing, Analysis and Visualization Software; Center for Information Technology, National Institutes of Health). Post-contrast images were then co-registered with 6 degrees of freedom to the corresponding pre-contrast images. Pre- and post-contrast images were then subtracted and window-levelled similarly.

## Results

In a span of one year, 47 patients who presented with suspected TBI were enrolled and imaged. Of the 47 patients screened for this analysis, 21 were excluded before the evaluation of T1WI pre- and post-contrast pairs and FLAIR pre- and post-contrast pairs (Fig 1). One subject was excluded after review of FLAIR image pairs revealed subdural and subarachnoid blood not initially detected by clinical staff. Demographics and presentation characteristics for the 25 subjects included in this analysis are detailed in Table 1.

**Table 1:** Clinical demographic characteristics of study population (N = 25).

**Age (Median, Interquartile Range (IQR))**	49 (35–62)
**Male**	15 (60.0%)
**Race**	
White	20 (80.0%)
African American	3 (12.0%)
Asian	1 (4.0%)
Other	1 (4.0%)
**Hispanic/ Latino**	4 (16.0%)
**Hours to MRI from Injury (median, IQR)**	6.07 (4.33–19.58)
**Glasgow Coma Scale (GCS)**	
15	22 (88.0%)
14	2 (8.0%)
13	1 (4.0%)
**Loss of Consciousness (LOC)**	13 (52%)
<1 minute	4 (31%)
1–29 minutes	9 (69%)
**Post Traumatic Amnesia (PTA)**	13 (52.0%)
1 second-10 minutes	5 (39.0%)
>10 minutes-30 minutes	1 (8.0%)
>30 minutes-1 hour	2 (15.0%)
>1 hour-12 hours	1 (8.0%)
>12 hours-24 hours	2 (15.0%)

Readers A and B had almost perfect agreement for TME on FLAIR based on a Kappa of 0.90. However, based on a Kappa of -0.24, readers A and B had disagreed more frequently than agreed upon presence of TME on T1WI. Average intra-rater agreement within the two sequence reads, showed Reader A to be more consistent and demonstrated almost perfect agreement with a kappa of 0.90. Reader B showed moderate agreement with a kappa of 0.48. Reader C performed a tiebreaker read for 13 of the T1WI and 1 of the FLAIR sequences for which readers A and B disagreed (Table 2).

**Table 2:** Breakdown of blinded scoring for presence of traumatic meningeal enhancement.

Subject	MRI Sequence					
	FLAIR			T1WI		
	Reader A	Reader B	Tiebreaker	Reader A	Reader B	Tiebreaker
1	0	0		0	0	
2	1	1		1	0	1
3	1	1		1	0	1
4	0	0		0	1	0
5	0	0		0	0	
6	0	0		0	0	
7	1	1		1	1	
8	0	0		0	0	
9	0	0		0	1	1
10	0	0		0	1	0
11	0	0		0	0	
12	0	0		0	1	0
13	0	0		0	1	0
14	1	1		1	0	1
15	0	0		0	1	0
16	0	0		0	0	
17	0	0		0	0	
18	0	0		0	1	0
19	1	1		1	0	0
20	0	0		0	0	
21	0	0		0	0	
22	1	1		1	0	0
23	0	1	1	0	0	
24	1	1		1	0	0
25	0	0		0	0	

0 = sequence read as negative; 1 = sequence read as positive.

**Fig 3:**
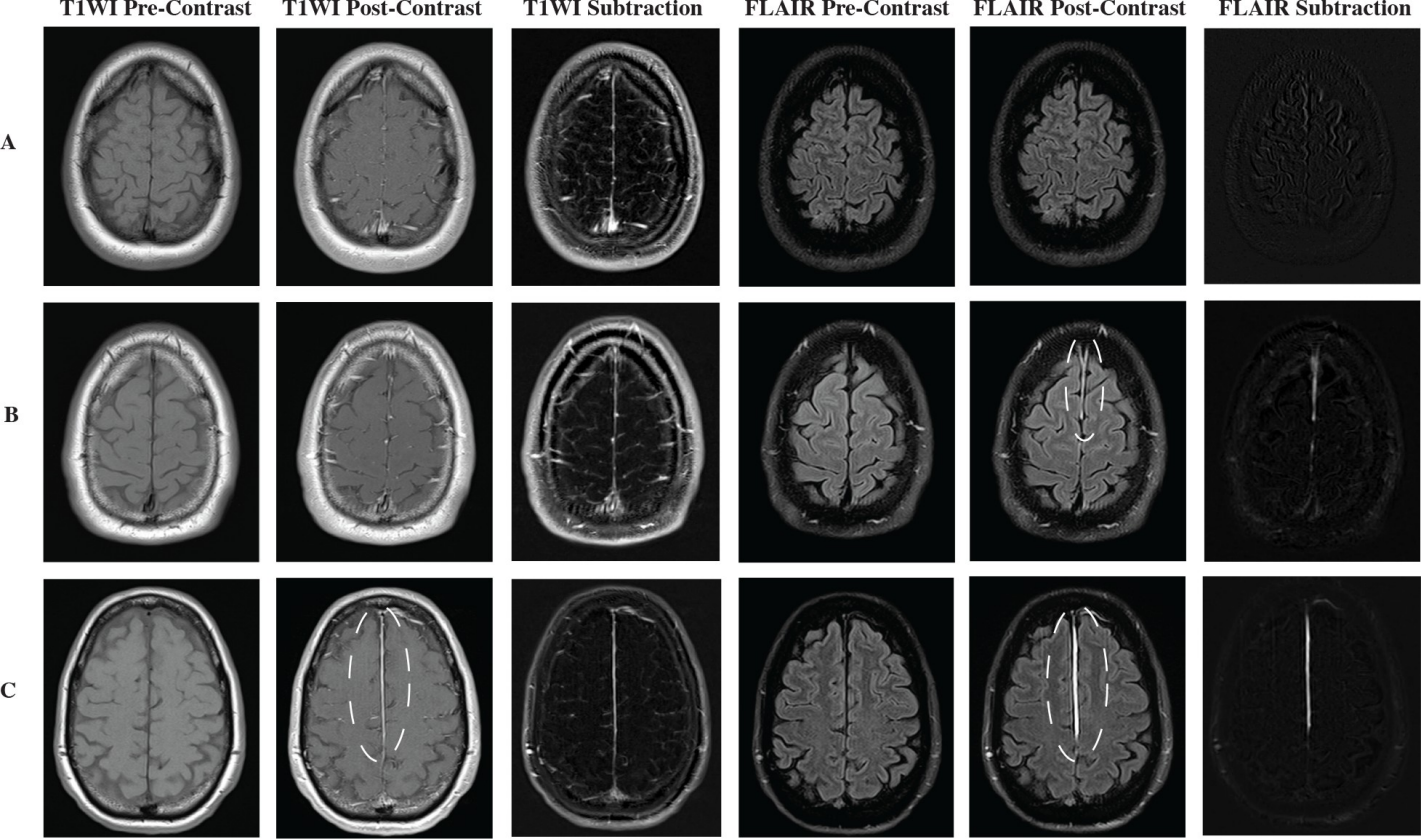
Examples of subjects studied in this cohort with possible scoring combinations by blinded readers. (A) Sets of T1WI and FLAIR pre and post-contrast of a subject scored as negative all around by all readers on both sets of sequences. (B) Sets of T1WI and FLAIR pre and post-contrast for a subject scored as positive for traumatic meningeal enhancement only on FLAIR sequence, as denoted by the dotted line. Traumatic meningeal enhancement is most conspicuous in the FLAIR post-contrast scan in both the falx cerebri and right convexity. (C) Pairs of T1WI and FLAIR pre and post-contrast scored as positive for traumatic meningeal enhancement on both sequences. Traumatic meningeal enhancement is denoted by the dotted line and is visualized in the falx cerebri alone on both post-contrast scans. The subtraction images visually represent the calculated differences between the pre-contrast and post-contrast T1 or FLAIR images, respectively. The T1 subtraction displays a vascular pattern not specific to meningeal enhancement, while the FLAIR subtraction primarily shows TME when it is present.

Fig 3A shows an example of paired pre- and post-contrast T1WI and FLAIR scans representing the 64% (16/25) of subjects that were negative for both TME and FLAIR. While there was high agreement by raters for positive findings of TME on post-contrast FLAIR, there was greater disagreement regarding findings on T1WI. Of the subset of patients who were positive for TME on FLAIR, 38% (3/8) were scored as negative on T1WI by the majority of readers (Fig 3B). 62% (5/8) of the patients who were positive on FLAIR for TME were scored as positive on T1WI (Fig 3C).

**Fig 4:**
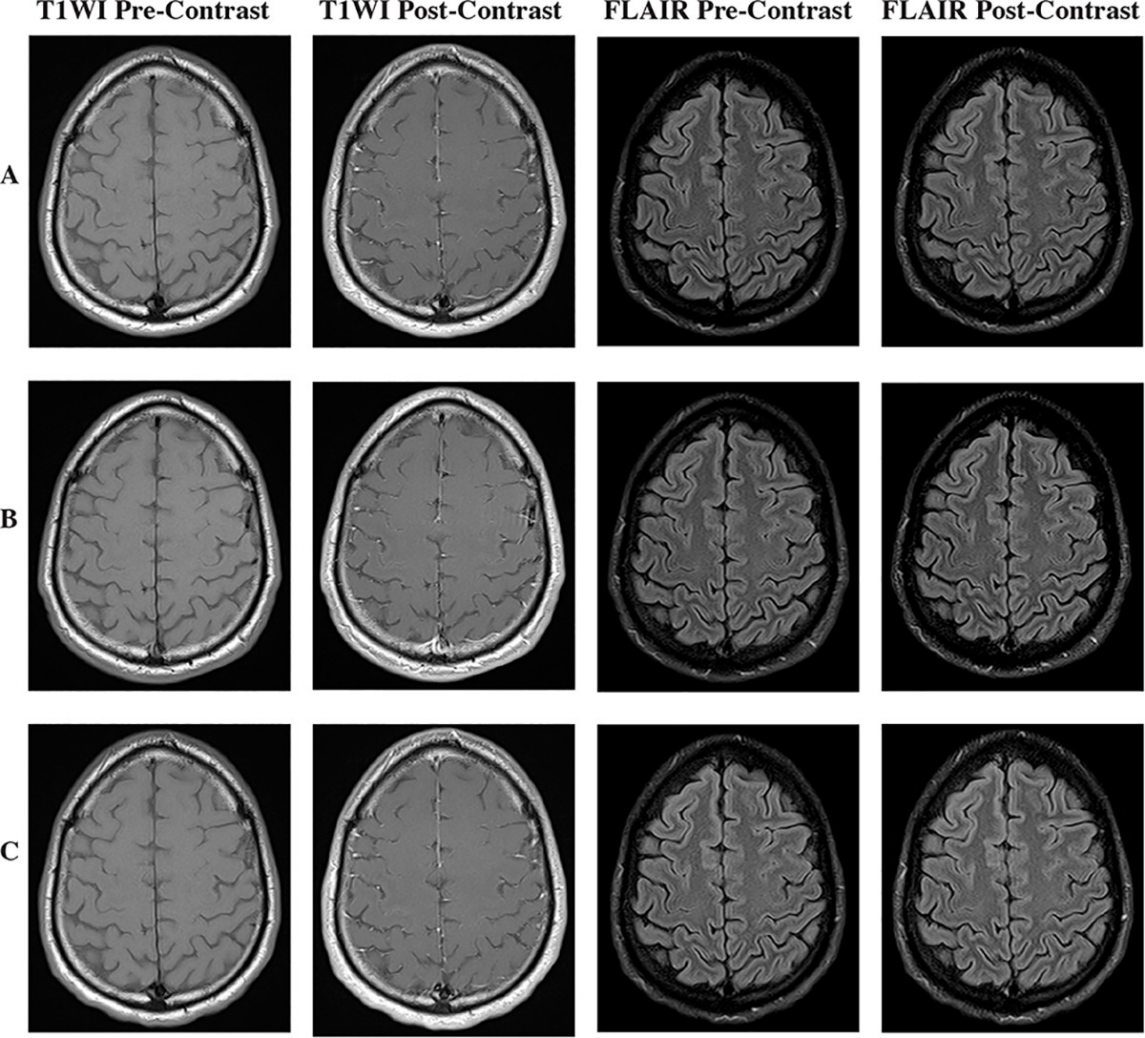
Imaging from the only subject scored positive for traumatic meningeal enhancement on T1WI post-contrast, but negative on FLAIR post-contrast. Pre-contrast T1WI and FLAIR images are shown for comparison. (A) According to raters this patient was scored positive due to the visualized pattern of contrast which seemed to thicken in the falx cerebri on the acute post-injury T1WI post-contrast sequences, while showing no findings on FLAIR post-contrast. (B) One week post-injury MRI shows a similar pattern of enhancement in falx cerebri-of T1WI post-contrast and a consistent lack of enhancement on FLAIR post-contrast. (C) T1WI and FLAIR post-contrast sequences one year post injury show once again a similar pattern of contrast on T1WI post-contrast sequence with no associated findings on FLAIR post-contrast sequence.

Fig 4 depicts the scans of the one subject that was scored as positive for TME only on post-contrast T1WI.

## Discussion

This study demonstrates that TME is detectable in some subjects on T1WI post-contrast; however FLAIR post-contrast is superior to T1WI post-contrast sequence to determine the presence or absence of TME. Both interrater agreement and overall conspicuity were higher for post-contrast FLAIR than for post-contrast T1WI. While 62% of subjects with TME were scored as positive on both types of post-contrast sequences, T1WI post-contrast fails to depict the novel imaging biomarker TME in 38% of subjects where TME can be readily seen on FLAIR post-contrast.

Only a single subject was scored as positive for TME on T1WI but not FLAIR. Longitudinal study of patients with TME indicates that the presence of TME as seen on post-contrast FLAIR decreases over time while post-contrast T1WI patterns remain unchanged [14]. For this subject, additional longitudinal images from the THINC study were available and were subsequently reviewed (Fig 4). This subject’s post-contrast T1WI findings did not change over later time points, indicating that this was likely a false positive.

Clinically, T1WI post-contrast sequences are preferred to visualize enhancement in hyperintensities seen with hypervascular pathologies (e.g., infection, neoplasia) or with contrast extravasation into the parenchyma after disruption of the blood-brain barrier along a blood vessel [12, 15]. The FLAIR sequence is a unique post-contrast sequence that has a longer repetition time (TR), echo time (TE) and inversion time (TI) than that of T1WI. The long TI in the FLAIR sequences produces a mild T1 effect which allows enhancement to be visualized on FLAIR post-contrast imaging [15, 16]. FLAIR post-contrast MRI is known to have higher sensitivity than T1WI post-contrast to depict disruption of the blood-CSF barrier [17, 18]. The higher conspicuity of FLAIR post-contrast MRI for detection of TME, as found in our study, is in accordance with the imaging findings in other disease states disrupting the blood-CSF barrier [19, 20].

Our demonstration that FLAIR post-contrast MRI is better than T1WI to detect TME in mild TBI patients imaged within 48 hours of injury is consistent with the results of a study of FLAIR post-contrast MRI within 14 days of mild TBI [11] that did not exclude patients with intracranial hemorrhage. Mild TBI patients with evidence of traumatic meningeal enhancement only on FLAIR post-contrast imaging have different patterns of peripheral blood gene expression within 48 hour of injury than mild TBI patients who have no evidence of TME or other intracranial injury [21]. TME likely represents damage to the meninges, with contrast leakage into the CSF as indicated by TME alone representing the mildest injury, and extra-axial hemorrhage demonstrating the more severe injury, as is suggested by findings of TME associated with subdural hematoma and/or subarachnoid hemorrhage [11].

While this study demonstrates that FLAIR post-contrast MRI is the better sequence for the depiction of TME, there were some limitations relating to this study. First, this analysis only comprised a small set of subjects with mTBI presenting at a single medical center. Second, the imaging protocol resulted in T1WI post-contrast MRI performed approximately 4 minutes after gadolinium administration while FLAIR post-contrast MRI was performed approximately 5 mins after contrast was given. An increased delay between gadolinium administration and T1WI MRI can result in larger contrast-enhancing volumes [22]. However, given the higher sensitivity of FLAIR MRI for a T1-shortening agent as compared to T1WI MRI, the results of this study would likely still hold even if the imaging order of the two sequences was reversed. Another limitation of the study was that our two radiology readers had different levels of expertise.

To demonstrate that TME could be rapidly detected on MRI in the acute clinical care setting (within 48 hours of injury), we focused here on the qualitative assessment of the presence or absence of TME. A more quantitative approach would include subtraction of pre- and post-contrast T1 and FLAIR images, as demonstrated in the subtraction images shown in Fig 3. Surface rendered 3D reconstructions, as seen in our prior publication [10], can also be used to display the extent of TME.

In this study we found that TME, a traumatic disruption in the blood-CSF barrier, is more conspicuous on FLAIR post-contrast. These findings are clinically important because TME is the most common MRI finding in mTBI patients who may otherwise not show any other lesion on either CT and/or MRI [8, 10, 14]. Whether or not TME is associated with clinical outcomes in mild TBI patients is an area of active investigation in our group. The inclusion of FLAIR post-contrast may be a valuable addition to clinical TBI imaging protocols.

## Conclusions

In conclusion, the conspicuity of TME is higher on FLAIR post-contrast MRI than T1WI post-contrast MRI. FLAIR post-contrast MRI is the preferred imaging technique for the detection of TME in TBI.
